# Open and hidden agendas of "asymptomatic" patients who request check-up exams

**DOI:** 10.1186/1471-2296-12-22

**Published:** 2011-04-19

**Authors:** Sabina Hunziker, Martin Schläpfer, Wolf Langewitz, Gilbert Kaufmann, Reto Nüesch, Edouard Battegay, Lukas U Zimmerli

**Affiliations:** 1 Medical Outpatient Division/Ambulatory Internal Medicine, University Hospital Basel, Petersgraben 4, 4031 Basel, Switzerland; 2 Division of Internal Medicine, University Hospital Zürich, Raemistrasse 100, 8091 Zürich, Switzerland; 3 Division of Psychosomatic Medicine, Department of Internal Medicine, University Hospital Basel, Petersgraben 4, 4031 Basel, Switzerland; 4 Division of Infectious Diseases, Hirslanden-Klinik St. Anna, St.-Anna-Strasse 32, 6006 Lucerne, Switzerland

## Abstract

**Background:**

Current guidelines for a check-up recommend routine screening not triggered by specific symptoms for some known risk factors and diseases in the general population. Patients' perceptions and expectations regarding a check-up exam may differ from these principles. However, quantitative and qualitative data about the discrepancy between patient- and provider expectations for this type of clinic consultation is lacking.

**Methods:**

For a year, we prospectively enrolled 66 patients who explicitly requested a "check-up" at our medical outpatient division. All patients actively denied upon prompting having any symptoms or specific health concerns at the time they made their appointment. All consultations were videotaped and analysed for information about spontaneously mentioned symptoms and reasons for the clinic consultation ("open agendas") and for cues to hidden patient agendas using the Roter interaction analysis system (RIAS).

**Results:**

All patients initially declared to be asymptomatic but this was ultimately the case in only 7 out of 66 patients. The remaining 59 patients spontaneously mentioned a mean of 4.2 ± 3.3 symptoms during their first consultation. In 23 patients a total of 31 hidden agendas were revealed. The primary categories for hidden agendas were health concerns, psychosocial concerns and the patient's concept of disease.

**Conclusions:**

The majority of patients requesting a general check-up tend to be motivated by specific symptoms and health concerns and are not "asymptomatic" patients who primarily come for preventive issues. Furthermore, physicians must be alert for possible hidden agendas, as one in three patients have one or more hidden reasons for requesting a check-up.

## Background

Check-up examinations, referred to as periodic health examination (PHE), or an annual physical examination, are among the most common reasons adults see a physician. From 2002 to 2004, approximately 44 millions US adults per year received a periodic health examination [1]. For European countries, epidemiological data on the use of check-up examinations have not yet been reported.

When patients request check-ups, physicians may assume it is for detection of asymptomatic disease. However, patients who request a check-up examination may expect more than just routine screening in accordance with current medical guidelines [2-4]. The actual reasons behind most check-up examinations requested by patients are generally unknown and qualitative data about the percentage of patients who request check-ups for reasons other than routine screening is lacking. Patients may openly mention their reasons to the physician ("open agenda"), or they might not ("hidden agenda"). Health maintenance is the declared objective of a check-up examination. Yet, often there may be hidden motives- to use the doctor as a counsellor to discuss problems, to seek reassurance regarding undeclared symptoms or to find security [5]. These aspects may be systematically ignored or overlooked in check-up consultations while the physicians tend to focus on delivering of screening recommendations. Most recommendations for PHE are based largely on the prevalence of preventable disease in asymptomatic individuals [6]. If patients who present for check-ups have other problems for which they are seeking care, the recommendations may be incomplete. In particular, hidden reasons for requesting an examination need to be deciphered in order to prevent patient dissatisfaction with the level of care that is provided, to the scheduling of unnecessary tests and follow-up consultations, or to the patients' symptoms being left unevaluated and untreated.

Even though a hidden agenda in communication is a widespread phenomenon throughout all specialities [7], little is known about its extent, especially in check-up patients. The main aim of this study was to evaluate which percentage of patients requesting a check-up exam has hidden agendas. We therefore prospectively evaluated self-declared asymptomatic patients requesting a check-up exam by analysing patient doctor communication and interaction for behavioural cues to hidden agendas.

## Methods

### Subjects and design

The study was conducted from December 2003 through November 2004 at the Medical Outpatient Division/Ambulatory Internal Medicine at the University Hospital Basel in Switzerland.

Consecutive participants were prospectively selected from patients who explicitly and spontaneously (without referral) requested a check-up exam at the time they scheduled their appointments with a receptionist and who neither mentioned having any symptoms nor requested a particular medical subspecialty, even when specifically prompted to provide such information by the administrative personnel who scheduled the appointments. Thus, all patients were self-referrals to the Medical Outpatient Division of the University Hospital Basel, Switzerland.

According to study protocol, all participants were scheduled for two consecutive patient consultations. First, a 45-minute baseline routine consultation with a resident and a supervising attending physician in the clinic, and second, a follow-up consultation 10-14 days later with a member of the research team. Both consultations were videotaped with the informed and written consent of the patient.

At the beginning of the baseline consultation, all patients were explicitly asked about their reasons for the clinic consultation. The initial consultation included taking a careful patient history, a complete physical examination, blood pressure measurement, and a laboratory assessment that included full blood count, biochemistry, and lipid panel. Everything during the first consultation, including the need for additional testing, was left to the discretion of the resident and attending physician with no input from the research team.

### Setting

In Switzerland, all patients have universal healthcare coverage, including adults with low income who receive social aid to cover healthcare costs, regardless of their age or whether they work. Patients are free to choose their primary care physician (GP) and can schedule appointments with specialists without referral being required.

The University Hospital Basel is a teaching hospital that provides primary, secondary, and tertiary care for a region with a population of approximately 200,000 people. The Medical Outpatient Division, which is open to the public without a referral being required, provides about 17,000 internal medicine consultations each year. These include about 3400 new consultations and around 8% of these being requests for a periodic health exam [8]. The medical staff consists of 11 residents in internal medicine, most of whom have completed more than four years of postgraduate clinical training, and 5 attending physicians who supervise them.

The study was approved by the local Ethics Committee (Ethikkommission beider Basel, EKBB 173/03).

### Definition of cue and hidden agenda

The Roter interaction analysis system (RIAS) [9,10] is a standardized coding system that is tailored to dyadic exchanges specific to a medical consultation. The entire patient-physician dialogue is coded into mutually exclusive categories that apply to each speaker. The method has demonstrated good reliability and predictive validity in studies of patient-physician communication [9-16].

In RIAS, a *cue *denotes an element in patient-provider communications that is not explicitly expressed verbally. It includes vague indications of emotions such as anxiety or embarrassment that patients might find difficult to express openly and that prevent the patient from being completely forthcoming about his or her reasons for requesting a consultation [17,18]. For the purpose of this study *cues *were also underlying unstated emotions, concerns, or expectations [19,20]. We defined a *cue *as a verbal or nonverbal hint that suggested an underlying unpleasant emotion, a patient's expectation regarding either a possible underlying disease that causes his symptoms or the outcome of the consultation. The following were classified as *cues*; unsolicited information given by the patient, a new element that was introduced into the conversation by the patient and directed the physician's attention to something that was worrying the patient or that had not yet been sufficiently discussed. If the patient repeated a subject that had already been addressed during the consultation, thus redirecting the physician's attention to it, this action was also coded as a *cue*. In addition, emotionally neutral statements by the patient that focused on issues of potential emotional importance or that referred to recent stressful life events, expectations, or concerns, were also coded as *cues*. Nonverbal signs (such as sighing, silence after provider questions, frowning, or crying) were defined as overt expressions of negative or unpleasant emotions or indications of hidden emotions.

The term *hidden agenda *refers to relevant information that the patient has either intentionally or unintentionally withheld [7,21-23]. A hidden agenda was assumed to exist if a patient confirmed in the follow-up appointment, that a cue contained a relevant- although previously unstated- concern or reason for the clinic visit.

### Analysis of patient interview videotapes

The videotape of the baseline consultation was independently analysed by two members of the research team. The team looked for verbal or nonverbal *cues *in the communication between the doctor and patient that might indicate a hidden patient agenda for requesting a check-up. When the findings of the two team members differed, the video was discussed with a third member of the study team and a decision regarding the behavioural *cues *was reached by consensus.

At the follow-up consultation the patient was explicitly asked about the verbal and nonverbal *cues *noted in the videotape by a research team member. If, after being confronted by the physician, the patient confirmed, that the cue contained a relevant, although previously unstated, concern or reason for the clinic visit, then the patient fulfilled criteria for a hidden agenda based on our outcome definition above. Two members of the research team also independently analysed the videotape of this follow-up consultation in order to confirm the presence or absence of a hidden agenda.

### Statistical analyses

The Kolmogorov-Smirnov test was used to assess for normal distribution of the data. Data was expressed as the mean ± standard deviation if the distribution was normal or as the median (interquartile range) if distribution was not normal. Categorical variables were presented as frequency counts. For continuous variables, differences between the groups were evaluated by using the unpaired Student's t-test for variables that were normally distributed or the Mann-Whitney U-test for variables that were not distributed normally.

All calculations were performed by using SPSS/PC (Version 16.0, SPSS Inc, Chicago, IL, USA).

## Results

**Figure 1:**
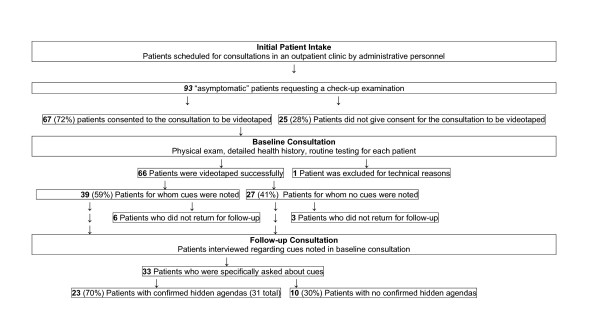
**Overview of results with respect to determining the presence of hidden patient agendas**.

The study setting and study participants' demographics are depicted in Figure 1 and table 1 respectively. A total of 93 patients met the inclusion criteria, of whom 25 (28%) patients did not give consent for the consultation to be videotaped and one patient was excluded for technical reasons. A total of 66 patients gave informed consent and their baseline consultation was videotaped successfully and analysed. Nine of the 66 patients (14%) were lost to follow-up. In 25 (38%) of the patients, the check-up examination revealed previously undiagnosed conditions (data not shown), most commonly mood disorders, including depression, and anxiety disorders, dyslipidemia, and hypertension.

**Table 1:** Baseline patient characteristics

Male/female (*n*)	43/23
Age (in years)	45 ± 16
Marital status: married/single (*n*)	43/23
Working (*n*)	34
Unemployed or Retired (*n*)	22
Student (*n*)	5
Receiving disability benefits (*n*)	5
Nationality:	
- Swiss (*n*)	34
- German (*n*)	7
- Italian (*n*)	7
- Turkish (*n*)	7
- Other (*n*)	11
Under the care of a GP* (*n*)	45

*GP = General Practitioner

Without any prompting from the physician during the baseline consultation 59 (89%) patients explicitly mentioned a mean of 4.2 ± 3.3 symptoms (range 1-15) during the check-up. Chest pain, back pain, dyspnea, nervousness, palpitations, and fatigue were the most frequently mentioned symptoms (data not shown). 45 of the 66 patients had a GP that they regularly consulted; 23 of the 45 patients with a GP had had regular appointments within 6 months prior to the baseline consultation.

At baseline consultation, the 66 patients explicitly gave a total of 206 specific reasons for requesting the exam. The most frequent motivations were (1) routine check-up to prevent illness, (2) diagnostic evaluation of specific symptoms, (3) laboratory testing or radiological imaging, (4) health concerns or fear of illness, and (5) cardiac examination. A detailed list of the patients' specific motives is provided in Table 2.

**Table 2:** Reasons for requesting a check-up that were mentioned during the baseline consultation ("open agenda")

Reason for requested check-up	Number of patients*
Prevention/general health exam	34
Diagnostic evaluation of specific symptoms	27
Blood tests or radiological tests	21
Health concerns/fear of illness	18
Cardiac examination	13
Evaluation of hypertension	8
Vertigo/dizziness	8
Sleeping disorders/fatigue	7
Life-style counseling (diet, smoking, surgical procedures, vaccination)	7
HIV^†^-Testing	7
Dyspnea/cough/wanted lung examination	6
Various other examinations	6
Sent by friends or relatives for a check-up	6
Urinalysis	5

*More than one reason was mentioned by most patients; ^†^HIV = Human immunodeficiency virus

Videotape analysis of the baseline consultation revealed a total of 51 verbal and nonverbal *cues *in 39 (59%) patients (Figure 1). *Cues *were classified into the following categories: (a) general health concerns (e.g. fear of cancer) and lifestyle issues (e.g. overweight) (n = 14), (b) patient's illness perception (e.g. a patient believes that his headache is caused by cerebral haemorrhage) (n = 15), (c) psychosocial concerns (e.g. financial problems) (n = 10), (d) worry about a specific illness (n = 8), and (e) illness in the social environment (e.g. a patient is scared of hepatitis because a family member got infected with hepatitis) (n = 5) (Table 3). Of the 33 patients who returned for the follow-up consultation and in whom *cues *had been noted in baseline consultation, 23 (70%) patients revealed one or more hidden agendas (a total of 31) when questioned about the verbal or nonverbal *cues *that we noted in the videotapes. In 10 patients, verbal or nonverbal *cues *that had been identified in the videotapes were not confirmed by the patients during the follow up consultations.

**Table 3:** Cues revealed by patients during baseline consultation, overall n = 39

CUE	Number of patients
Patient's illness perception	15
Psychosocial concerns	10
Worried about a specific illness	8
Illness in the social environment	5
Health concerns	
- HIV*	4
- Cancer	3
- Heart disease	3
- Lifestyle issues (smoking, diet, drinking)	3
- Hypertension	1

*HIV = Human immunodeficiency virus

The main primary categories for hidden agendas were health concerns and lifestyle issues (n = 14), psychosocial concerns (n = 8), and the patient's concept of disease (n = 6) (Table 4).

**Table 4:** Hidden agendas revealed by patients during second consultation, overall n = 23

Hidden agenda	Number of patients
Psychosocial concerns	8
Patient's concept of disease	6
Illness in the social environment	3
Health concerns:	
- Cancer	4
- HIV*	3
- Heart Disease	3
- Lifestyle (smoking, diet, drinking)	2
- Hypertension	2

*HIV = Human immunodeficiency virus

Baseline characteristics of patients for whom *cues *were noted (n = 39) and those for whom they were not noted (n = 27) were not different in terms of gender (69% *vs*. 59% male, *P *= 0.44), mean age (47 *vs*. 41 years of age, *P *= 0.19), marital status (69% *vs*. 70% married, *P *= 1.00), employment status (51% *vs*. 48% employed, *P *= 0.80), or nationality (54% non-Swiss citizens *vs*. 44% Swiss citizens, *P *= 0.62). Furthermore, there was no significant difference in terms of the mean number of declared symptoms (4.5 ± 3.6 *vs*. 3.8 ± 2.9; *P *= 0.48) and reasons for requesting the exam (3.3 ± 1.8 *vs*. 2.8 ± 2.2; *P *= 0.13).

Baseline characteristics of patients with an identified underlying hidden agenda (n = 23) and patients without an identified hidden agenda (n = 10) also did not differ (age: 49 *vs*. 44 years, *P *= 0.20; gender: 65% *vs*. 64% male, *P *= 0.99; marital status: 61% *vs*. 76% married, *P *= 0.25; employment status 43% *vs*. 53% employed, *P *= 0.59; nationality: 52% non-Swiss citizens *vs*. 50% Swiss citizens, *P *= 0.87).

The inter-rater reliability for cues in the baseline consultation and hidden agenda in the follow-up consultation was acceptable (Cohen's Kappa 0.76 and 0.82, respectively).

## Discussion

We have found that the majority of patients requesting a check-up exam expect more than just routine screening. Patients had a variety of specific complaints and symptoms that were explicitly verbalized during baseline consultation. Furthermore, using a standardized technique (RIAS) to analyze verbal and nonverbal interaction of patients and physicians, *cues *were often noted in the videotapes of the consultations. When specifically addressing these in the follow-up consultations with the patients, hidden agendas, such as health concerns and psychosocial problems, were found in a third of the patients. These might actually be the primary reasons for patients' requests for check-ups.

A check-up is an important opportunity for counseling patients about specific disease-related issues and for early detection of (asymptomatic) disease. Our data shows that patients are rarely asymptomatic and that they rarely request a check-up for the purpose of routine screening for disease prevention. During the baseline consultation, only half the patients indicated an "unspecific general health examination" as the main reason for the consultation, even though all patients had asked for a "routine check-up" at the time they scheduled their appointments. Moreover, the majority of these apparently "asymptomatic" patients spontaneously mentioned a variety of symptoms and specific complaints when interviewed during the initial baseline consultation. The variety of symptoms resulted in a high number of diagnostic tests; most of them not recommended by screening guidelines [6]. Obviously, symptoms had induced concerns in the patients about the possibility of an underlying disease.

In this study, 41% of patients specifically requested clarification of a particular symptom as their main motivation for requesting the check-up. This is in accordance with the finding that the public beliefs it to be important to remain in "good health", and that this is an important stimulus for requesting a check-up [2,24]. A systematic review by Boulware et al has demonstrated that the PHE has a beneficial effect on the delivery of some clinical preventive services and may have a beneficial effect on patient worry, providing justification for its continued implementation in clinical practice [25].

Hypothetically, worries and symptoms that have not been explicitly mentioned by patients can have a great impact on their decisions to request check-ups [21,26]. When their expectations of care are not met, it is an important factor in lower patient satisfaction, which, in turn, is associated with less adherence to therapy, more health care utilization, more frequent malpractice litigation, and switching doctors or health plans [27-31]. Therefore, recognizing a patient's true concerns and worries and being able to evaluate both open and hidden patient agendas is important. Recognizing and identifying a hidden agenda is a challenging task. Being alert to behavioural and verbal *cues *is the road to success. The analysis of the communication in the baseline consultation uncovered *cues *in 59% of the patients. By discussing these *cues *with the patients during the follow-up consultations, we found that one in three patients had one or more hidden reasons for requesting a check-up. Neither demographic patient characteristics nor the quantity or quality of *cues *were indicative of hidden agendas. Nevertheless, a *cue *can identified during consultation warrants further exploration.

### Limitation of the study

Although two-thirds of our study participants had regular consultations with their primary care physicians, they still considered it necessary to schedule an additional appointment for a check-up at our internal medicine outpatient division after direct access without referral. Possibly, they did so because the university hospital provides all the diagnostic facilities available on-site. In particular, this might be the reason many patients who wished diagnostic procedures requested an appointment in a tertiary care outpatient clinic. Therefore we encourage future studies in the primary care setting in order to validate the generalisability of our findings.

The lack of a gold standard for defining a hidden agenda is another possible limitation of this study. We may have missed a number of underlying concerns that were not indicated by *cues *identified in the videotapes. We may therefore have underestimated the prevalence of hidden agendas. However, we did include several procedures to minimize errors by videotaping the consultations to allow more than just real-time analysis, having the tapes independently analysed by two team members, and including an option for additional analysis with a third team member if the results of analysis between the original two raters differed. Furthermore, this small study may not have had the power to show a difference between groups with and without *cues *and hidden agenda, respectively.

## Conclusions

This study shows that the majority of patients has a different semantic understanding than the health care system of what check-up exams means. Patients are rather motivated by a variety of symptoms to request an examination than by the concept of periodic screening to detect disease in its asymptomatic phase. Some symptoms and concerns are stated openly by the patient once they are talking directly with the physician. However, one in three patients had one or more hidden agendas for requesting a check-up. Therefore physicians need to be aware of possible *cues *to reveal hidden reasons for the consultation.

## Competing interests

The authors declare that they have no competing interests.

## Authors' contributions

SH and MS participated in data collection, analysis, and interpretation and in the writing of the manuscript. LZ, EB, and WL participated in the design of the study, in data collection, analysis, and interpretation, and in the writing of the manuscript. GK and RN participated in analysis, and interpretation, and in the writing of the manuscript. All authors approved the final version.

## Pre-publication history

The pre-publication history for this paper can be accessed here:

http://www.biomedcentral.com/1471-2296/12/22/prepub
